# Retraction Note: Silencing of Histone Deacetylase 9 Expression in Podocytes Attenuates Kidney Injury in Diabetic Nephropathy

**DOI:** 10.1038/s41598-024-58326-x

**Published:** 2024-04-02

**Authors:** Feng Liu, Ming Zong, Xiaofei Wen, Xuezhu Li, Jun Wang, Yi Wang, Wei Jiang, Xiaojun Li, Zhongliang Guo, Hualin Qi

**Affiliations:** 1 Department of Nephrology, Shanghai East Hospital, Tongji University School of Medicine, Shanghai, China; 2 Department of Laboratory, Shanghai East Hospital, Tongji University School of Medicine, Shanghai, China; 3 Department of Urology, Shanghai East Hospital, Tongji University School of Medicine, Shanghai, China; 4 Department of Respiratory, Shanghai East Hospital, Tongji University School of Medicine, Shanghai, China

Retraction of: *Scientific Reports* 10.1038/srep33676, published online 16 September 2016

The Editors have retracted this Article. Concerns were raised regarding a number of figures, specifically:

- The panel labelled HDAC9 of Figure 1C appears to be a duplicate of the panel labelled TRIM65 of Figure 1E of^1^;
- The panel labelled GAPDH of Figure 2 appears to be a duplicate of the panel labelled GAPDH of Figure 5D of^2^;
- The panel labelled STAT3 of Figure 5A appears to be a duplicate of the panel labelled Smad2 of Figure 5B of^3^;

The Authors were unable to provide the original data for examination. The Editors therefore no longer have confidence in the results and conclusions of this Article.

Feng Liu, Ming Zong, Zhongliang Guo, and Hualin Qi agree to this retraction. Xiaofei Wen and Yi Wang did not reply to correspondence from the Editors. The Editors were unable to contact Xuezhu Li, Jun Wang, Wei Jiang, and Xiaojun Li.
